# Harbour porpoises respond to recreational boats by speeding up and moving away from the boat path

**DOI:** 10.1002/ece3.11433

**Published:** 2024-05-15

**Authors:** Xiuqing Hao, Héloïse Hamel, Céline Hagerup Grandjean, Ivan Fedutin, Magnus Wahlberg, Caitlin Kim Frankish, Jacob Nabe‐Nielsen

**Affiliations:** ^1^ Department of Ecoscience Aarhus University Roskilde Denmark; ^2^ Department of Biology University of Southern Denmark Odense Denmark; ^3^ Fjord&Bælt Kerteminde Denmark; ^4^ Norwegian Polar Institute Tromsø Norway

**Keywords:** behavioural response, boat disturbance, drone footage, motorboat, *Phocoena phocoena*, recreational vessels, underwater noise

## Abstract

Recreational boats are common in many coastal waters, yet their effects on cetaceans and other sensitive marine species remain poorly understood. To address this knowledge gap, we used drone video footage recorded from a recreational boat to quantify how harbour porpoises (*Phocoena phocoena*) responded to the boat approaching at different speeds (10 or 20 knots). Furthermore, we used a hydrophone to record boat noise levels at full bandwidth (0.1–150 kHz) and at the 1/3 octave 16 kHz frequency band for both experimental speeds. The experiments were carried out in shallow waters near Funen, Denmark (55.51° N, 10.79° E) between July and September 2022. Porpoises were more likely to move further away from the path of the boat when approached at 10 knots, but not when approached at 20 knots. In contrast, they swam faster when approached at 20 knots, but not when approached at 10 knots. The recorded received sound level did not depend on how fast the boat approached, suggesting that differences in porpoise responses were related to the speed of the approaching boat rather than to sound intensity. In addition, porpoises generally reacted within close proximity (<200 m) to the approaching boat and quickly (<50 s) resumed their natural behaviour once the boat had passed, indicating that the direct impact of small vessels on porpoise behaviour was most likely small. Nevertheless, repeated exposure to noise from small vessels may influence porpoises' activity or energy budget, and cause them to relocate from disturbed areas. The approach used in this study increases our understanding of recreational boats' impact on harbour porpoises and can be used to inform efficient mitigation measures to help focus conservation efforts.

## Graphical abstract

We quantified harbour porpoises' responses to a motorboat moving at either 10 or 20 knots. Porpoises changed behaviour when the boat approached but they reacted differently to the approaching boat at different speeds. Porpoises appeared to be sensitive to how fast the noise level changed.

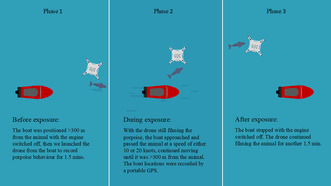

## 1 INTRODUCTION

As recreational boats become more prevalent in coastal waters worldwide, they increasingly interfere with wildlife (Carreño & Lloret, 2021; Davenport & Davenport, 2006; Hermannsen et al., 2019). In particular, species that use sound for foraging, navigating, and communicating, such as the harbour porpoise (*Phocoena phocoena*), are at risk of being disturbed. Vessel traffic is known to affect porpoise behaviour (Dyndo et al., 2015; Frankish et al., 2023; Wisniewska et al., 2018) and can potentially influence the animals' foraging success, fitness and population dynamics (Lusseau et al., 2023; Oakley et al., 2017; Wisniewska et al., 2018). However, studies investigating how animals react to recreational boats and how long their responses last are scarce. Considering the overlap of recreational boat traffic with harbour porpoise habitats (Hao & Nabe‐Nielsen, 2023) and the overlap between the frequency range of boat noise and porpoise hearing (Hermannsen et al., 2019), such knowledge is important for improving the conservation of porpoises and other cetaceans.

Cetaceans vary considerably in how they respond to vessel disturbance. Some species exhibit horizontal avoidance (Lemon et al., 2006; Marley et al., 2017; Nowacek et al., 2001), erratic movements (Bejder et al., 2006), increased swimming speed (Nowacek et al., 2001), or longer dive durations (Frankish et al., 2023; Wisniewska et al., 2018) when confronted by vessels. In some cases, vessels interrupt the animals' foraging behaviour (Wisniewska et al., 2018) or induce avoidance behaviour over distances >1 km (Palka & Hammond, 2001). However, some studies report that animals do not change behaviour, or even that they are attracted to vessels (Failla et al., 2004; Mesnick et al., 2002). Due to this variability in reactions, it is crucial to get a better understanding of when vessels start affecting cetacean behaviour, and how much. This is challenging, as it is difficult to measure the exact distance between animals and boats, and to observe changes in animal behaviour from a distance.

Over the past few years, the development of increasingly advanced drones (Unmanned Aerial Systems or UAS) has made it easier to observe cetacean behaviours remotely and non‐invasively (Álvarez‐González et al., 2023; Nowacek et al., 2016; Rees et al., 2018; Sprogis et al., 2020). Compared to traditional observations from boats or from land, drones have the advantage that they can hover over an animal while continuously collecting high‐quality data (Koh & Wich, 2012; Morimura & Mori, 2019; Rees et al., 2018). They also make it possible to quantify detailed behavioural changes and how these are related to features in the environment, including the distance to a boat (Chabot & Bird, 2015; Koh & Wich, 2012). With these advantages, drones hold great potential for enhancing our understanding of how anthropogenic disturbances affect marine animals like harbour porpoises.

In this study, we used a drone to quantify behavioural changes in harbour porpoises as they were approached by a recreational boat at a constant speed (either 10 or 20 knots). Based on the findings described above, we hypothesised that in response to the boat, porpoises would speed up, move away from the boat's path, execute large turns, dive deep, and decrease the likelihood of breathing. We also hypothesised that porpoises would respond more strongly to fast boats than to slow boats, as we speculated faster vessels would produce more noise, and be thus more disturbing. Furthermore, we compared porpoise behaviour while the boat was nearby (i.e., during the minute where the boat was closest) with their natural behaviour (i.e., prior to boat approach) and explored how rapidly porpoises resumed their natural behaviour, to assess if recreational boats are likely to have long‐term effects on porpoises. We measured the sound level at different distances from the boat to determine if porpoise responses were mostly related to the sound level or to the speed at which the boat approached. As porpoises are strictly protected in European waters (Council Directive 92/43/EEC, 1992), studies of how animals react to recreational boats, like the present study, are important for informing this species' management.

## 2 MATERIALS AND METHODS

### 2.1 Study site and experimental design

**FIGURE 1:**
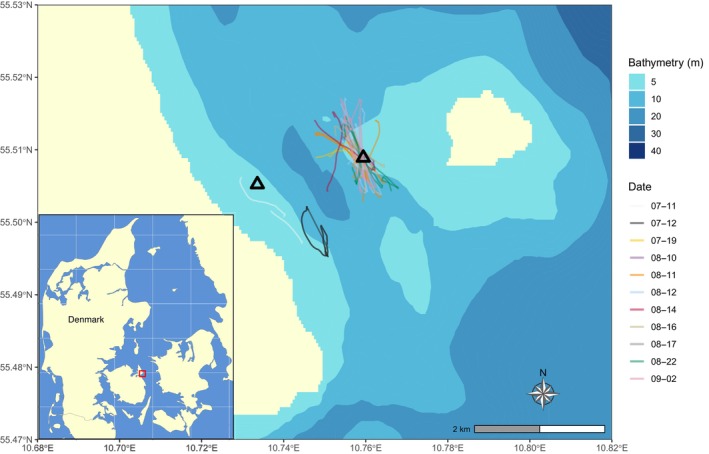
Area of boat disturbance experiments on harbour porpoises. Coloured lines show boat tracks on different dates. The black triangles represent the locations of the hydrophone used to record boat noise independently of the porpoise exposure experiments. The country map was extracted using the “rnaturalearth” package for r (Massicotte & South, 2023). Bathymetry data were obtained from https://emodnet.ec.europa.eu/en/bathymetry.

To investigate how harbour porpoises responded to approaching recreational boats, we conducted experiments using a research boat while monitoring porpoise movements with a DJI Phantom 4 Pro v2.0™ drone with a mounted camera recording in 4 K resolution (4096 × 2160 pixels) and up to 60 frames per second. The camera was equipped with a polarising filter to avoid sun glare in the video footage. The drone operator was on board during each experiment. The experiments were carried out in Romsø Sound, located by the eastern coast of Funen, Denmark (55.51° N, 10.79° E; Figure 1), which is an important habitat for harbour porpoises (Sveegaard et al., 2011) and an area with dense boat traffic during summer months. The experiments took place between 11th July and 10th September 2022, with a total of 20 days spent in the field collecting data, but porpoises were not sighted or recorded every day due to poor weather conditions on some (see Figure 1 for dates when videos were recorded). We conducted experiments on days with favourable weather conditions, i.e., wave height ≤0.3 m, wind speed <10 m/s, and without rain. It was done at water depths between 1 and 7 m to ensure that we could track the porpoise in the drone footage. The research boat used in the experiments was a 5.5 m Pioner Multi III, powered by an 80 hp outboard engine. Boat tracks were collected using a portable GPS (Garmin GPSMAP 78s). Drones flying at low altitudes (10–23 m) have minimal impact on cetacean behaviours (Aubin et al., 2023; Fettermann et al., 2019; Ramos et al., 2018), and when flown above 5 m, they have negligible effects on underwater noise levels (Christiansen et al., 2016). In our experiments, the drone was kept at an altitude of 10–30 m to minimise its disturbance to porpoises, while also flying low enough to keep track of animals when they were diving deep. We did not observe obvious reactions from porpoises to the drone. Our selection of 10 and 20 knots as experimental speeds was based on the observed travelling speed for motorboats equipped with outboard engines (without sails) in Danish waters; 10 knots corresponds to mean travel speed while 20 knots corresponds to fast moving vessels (Hao & Nabe‐Nielsen, 2023).

**FIGURE 2:**
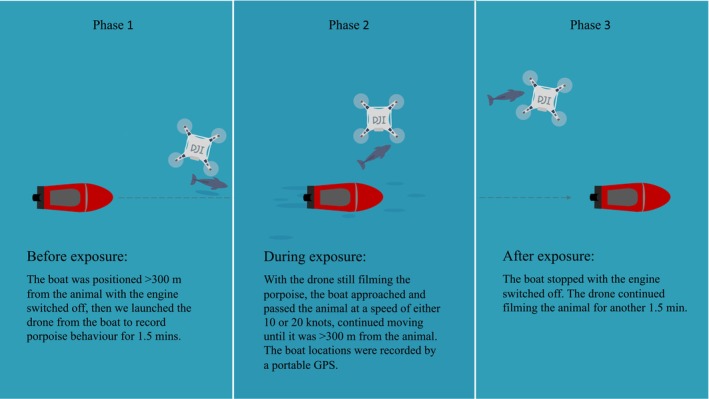
Experimental setup. The boat is only visible in the videos at the closest point of approach (CPA).

Each experiment consisted of three phases (Figure 2). (1) Upon sighting porpoises we positioned the research boat >300 m from any porpoise with the engine turned off to avoid any disturbance. For the 20 knots experiments, this distance was extended to >400 m to ensure porpoises had as much time to react to the boat as in the 10 knot experiments. Then we launched the drone from the boat to record their behaviour for 1.5 min. We only analysed data from the last minute of this phase when comparing with data collected during the exposure phase. Porpoises are unlikely to react to boats that are >250 m away (Baş et al., 2017; Oakley et al., 2017), which was the reason we positioned the boat >300 m from the porpoise. (2) With the drone still filming the porpoise, we started the boat and gradually increased the speed to its maximum (10 or 20 knots) over a period of 30 s. We passed the porpoise without changing direction and continued moving until the boat was >300 m away from the porpoise. Due to variations in currents and waves, the maximum speed (10 or 20 knots) varied by up to 2 knots among trials. We aimed to pass the target porpoise at a distance of 25 m. If the porpoise was diving, we estimated its location based on the position of the drone. When comparing behaviour in phase 1 and 2 we used data from the 1 min centred around the closest point of approach (CPA) of phase 2 and from the last minute of phase 1. (3) We turned off the engine and continued observing the porpoise for another 1.5 minutes with the drone. To limit the influence of external variables on porpoise behaviour, we only conducted experiments when there were no moving vessels <1 km from the porpoise. Each group of porpoises was only approached once, and we focused on one animal in each trial. After each trial, we moved more than 1 km away and waited for at least 30 min before approaching another porpoise to minimise the risk of exposing the same animal twice. The echosounder of the boat was switched off during the experiment. The potential harm to porpoise individuals was very limited, as animals only had a risk of being disturbed when the boat was moving, i.e., typically for 4–7 min.

After finishing the porpoise exposure experiments, we measured boat noise levels at two specific sites within the same area (Figure 1), where the water depth was about 5–6 m. Measurements took place under weather conditions identical to those during the exposure experiments. We suspended a stationary recorder (Sound trap, OceanInstruments, New Zealand; 576 kHz sampling rate, 16 bits, clipping level 176 dB re 1 μPa p, as determined by relative calibration) at either 1.5 or 2.7 m below the surface and attached it to a buoy. Just like in the exposure experiment, we approached the recorder at speeds of either 10 or 20 knots from various directions, starting from a distance of >300 m from the recorder. The boat was stopped when it was >300 m away from the recorder. This process was repeated five times for each boat speed at each site. The boat's geographical coordinates were recorded using a portable GPS (Garmin GPSMAP 78s).

### 2.2 Data handling

**FIGURE 3:**
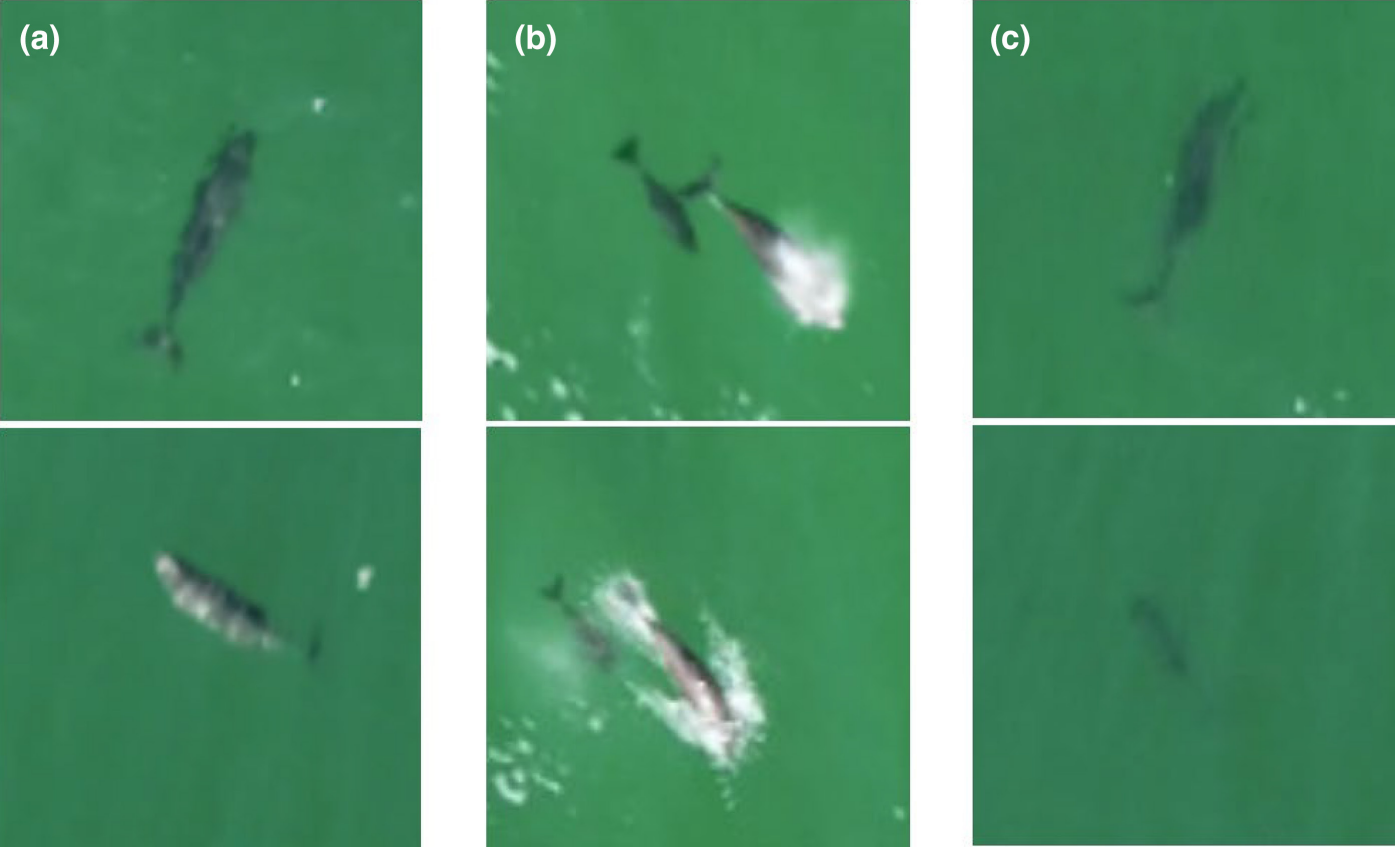
Examples of porpoise swimming states. (a) Shallow dive, non‐breathing; (b) shallow dive, breathing; (c) deep dive. Images were obtained from zoomed‐in drone videos.

**FIGURE A1:**
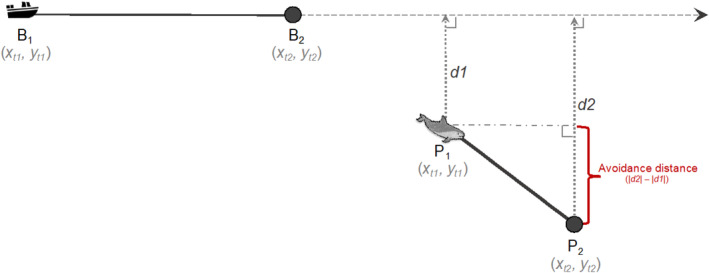
Calculation of a porpoise's reaction to an approaching boat at time t1. If avoidance distance >0, the porpoise is avoiding the boat. If we assume the porpoise remains neutral to boat disturbance, the avoidance distance from the boat track would be around 0, and the probability of moving away from the boat track is close to 0.5.

We used the Drone Video Measure tool (Version 1.1.1; Egemose, 2021) to extract position and swimming state of the target porpoise (one position per second). The swimming state was categorised as either shallow‐dive (porpoise body shape clearly visible; including breathing animals) or deep‐dive (porpoise body shape not clearly visible; Figure 3). To calculate the porpoise's speed and horizontal turning angles between successive moves, we applied the “adehabitatLT” package (Calenge, 2006) to porpoise locations (one per second). To quantify whether an animal tried to avoid the boat and its tendency to move away from the boat track, we calculated the distance between the boat track and the porpoise at two successive boat locations (i.e., 1 s apart; Figure A1 in Appendix 1). To ensure that porpoise tracks were temporally aligned with the boat tracks we compared the clocks in the boat GPS and in the drone at the point where the boat became visible in the drone video footage. When needed, we calibrated the drone clock based on the time difference between the GPS's. The location measurement accuracy was 2.4 ± 1.5 m when the drone was 30 m above the porpoise (Brennecke et al., 2022).

Data where the porpoise was lost in the video footage for more than 5 s during boat approaches (i.e. phase 2 before CPA) were excluded from all analyses. Thus, out of 27 recorded videos, only 17 (8 for boats moving at 10 knots; 9 for 20 knots) were used for analysing how porpoises changed behaviour as the boat approached, but 16 (7 for boats moving at 10 knots; the porpoise was lost in one of the experiments after boat approach; 9 for 20 knots) videos were used to determine if the animals' behaviour (speed, turning angle, the likelihood of deep diving and of breathing) during exposure (1 min around CPA) differed from their pre‐exposure behaviour, and to explore how long it took porpoises to return to their natural behaviour after the disturbance.

### 2.3 Data analysis

**FIGURE A2:**
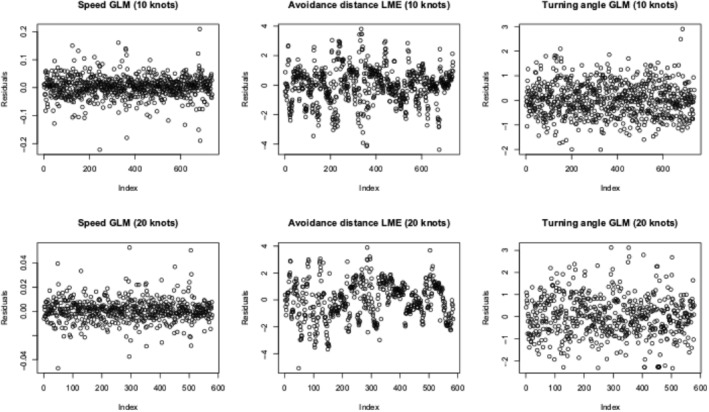
Model residuals for porpoises' behaviours in relation to their distance to the boat. Speed and absolute turning angles were transformed to their cube root form with a Gamma distribution; avoidance distance was modelled with a Gaussian distribution.

To analyse how porpoises responded to the approaching boat (i.e., phase 2 before CPA), we built six models for each boat speed, with either porpoise movement speed, change in distance from boat path (i.e., avoidance distance; Figure A1), probability of moving away from the boat path, absolute turning angle, probability of diving deep or breathing as a response variable. In all models we used log_10_(distance) as the independent variable, as sound levels are generally proportional to the logarithm of the distance to the sound source. Individual ID was included as a random effect, and an AR1 model (i.e. autoregression model at an order of 1) was used to account for temporal autocorrelation. To determine whether porpoises altered their speeds or absolute turning angles when the boat approached, we used generalised linear mixed effects models (GLMMs) with each behaviour as a Gamma distributed response variable. Porpoise speeds and absolute turning angles were cube root transformed in all statistical analyses to ensure that residuals were approximately normally distributed (see Figure A2 for model residual distribution). To determine how much porpoises moved away from the boat path as the boat approached, we used a linear mixed effects model (LME) with avoidance distance to the boat path (Figure A1) as the response variable. To examine how the probability that porpoises were avoiding the boat path, diving deep, or breathing less depended on distance to the boat, we built three GLMMs with each behaviour as a binary response variable. We used the Newey‐West variance estimator (by adding “sandwich” argument) to re‐estimate standard errors and associated significance levels (Newey & West, 1986), which accounts for autocorrelation between observations by inflating estimated standard errors (Lennon, 1999). To estimate the uncertainty of model predictions, we calculated 95% confidence intervals (CI) for each model. We used the Wilson score interval (Wilson, 1927) for probabilities associated with avoiding the boat, diving deep and breathing, because it had better coverage probability for binomial proportions (Brown et al., 2001).

To determine if porpoises responded more negatively to the boat approached at 20 knots than at 10 knots, we used the same methods and model types as described above for each behaviour, except that we included boat speed (categorical) and the interaction between boat speed and log_10_(distance) in the models.

To evaluate whether porpoises changed their behaviours during boat exposure compared with before exposure, we constructed GLMMs with experiment phase (before/during exposure; categorical) as independent variable and individual ID as a random effect. We accounted for temporal autocorrelation using the same method as above. To assess whether porpoises moved faster or turned more abruptly, we used GLMMs with either porpoise speed or absolute turning angle (both in their cube root forms with Gamma distribution) as response variables. To test whether the probability of diving deep or breathing was higher, we fitted binomial models. We fitted separate models for the 10 and 20 knot experiments. We used the “nlme” package (Pinheiro et al., 2007) to fit all LME models, and the “glmmTMB” and “glmmAdaptive” packages (Brooks et al., 2017; Rizopoulos, 2022) to fit all GLMMs.

To assess how long it took porpoises to resume their pre‐disturbance behaviour, we used generalised additive models (GAMs) with either porpoise speed, absolute turning angle, probability of diving deep or of breathing as response variables and time relative to the CPA as predictor (*H*_0_: the independent variables have no effect on the response). Individual ID was included as random effect (bs = “re”). Models were fitted using a Gamma distribution for speed or turning angle. For the probability of diving deep and of breathing, we used a binomial distribution. A *k*‐value of 5 for the smooth term was chosen to limit the risk of model overfitting. We fitted GAMs using the “mgcv” package for R (Wood, 2017).

We used one‐tailed tests to compute the statistical significance (i.e., *p* < .025 is of significance) for models evaluating how porpoises responded to the approaching boat. We estimated the proportion of variance in the response variables attributed to the independent variables by computing both marginal *R*‐Squared ($R_{m}^{2}$: variance explained by only fixed factors) and conditional *R*‐Squared ($R_{c}^{2}$: variance explained by both fixed and random factors). The boat passed the porpoises at an average distance of 26 m (range: 9–40 m) during the 10 knots experiment and at an average distance of 22 m (range: 4–55 m) during the 20 knots experiment.

To investigate how received noise levels were related to distance to the research boat, we used MATLAB (version 2022b) to analyse the recorded data. Noise levels (in dB re 1 μPa rms, 1 s average) were calculated at full bandwidth (0.1–150 kHz) and at the One‐Third Octave (TOL) 16 kHz frequency band. To investigate how noise levels changed over time for the two boat speeds, we calculated noise increments per 10 s for both frequency bands.

## 3 RESULTS

### 3.1 Porpoises' behavioural response to an approaching boat

**FIGURE 4:**
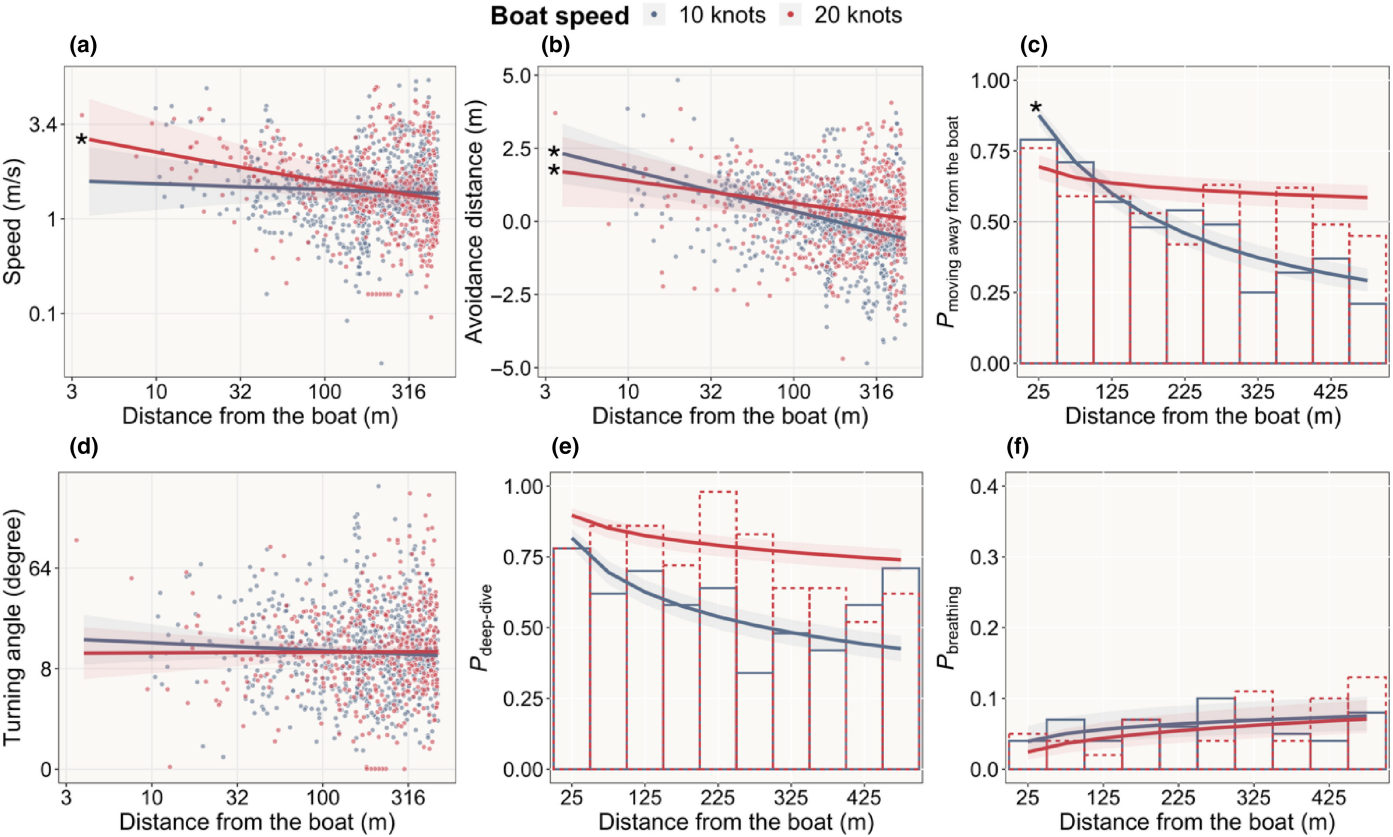
Observations (dots; a, b and d) and model estimates (lines) of porpoise behavioural responses to an approaching boat (averaged across animals; data from phase 2 before the closest point of approach, i.e., CPA). In sub‐figures (c), (e) and (f), bars show observed frequencies of a specific behaviour at each distance range. Shaded areas show 95% confidence intervals. * denotes significant models (*p* < .025).

**FIGURE A3:**
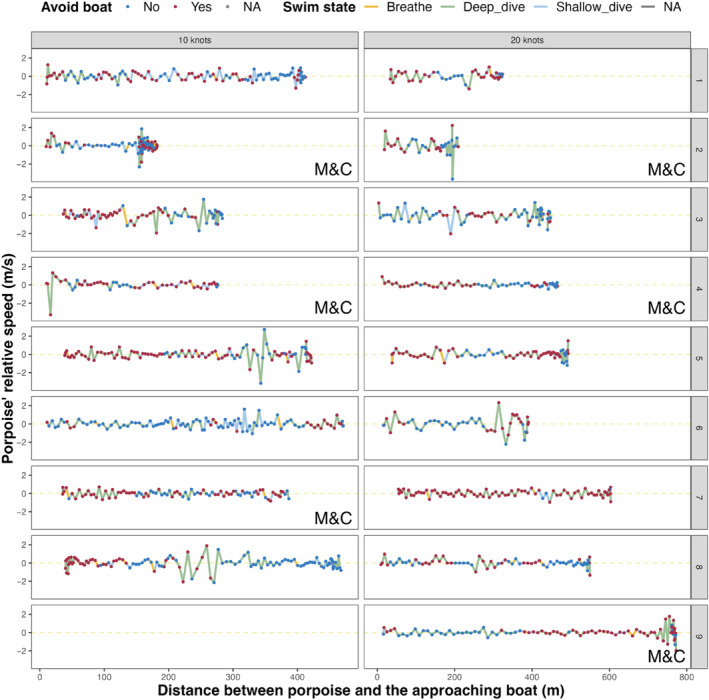
Individual porpoise's behaviour when a boat approached from when it started moving (at per second interval) to before the closest point of approach (CPA) at 10/20 knots. Porpoise's relative speed is the difference between speed at *T*_*t*+1_ and *T*_*t*_. M&C indicates mother‐calf pair was involved in the corresponding experiment.

A small motorboat approaching at 20 knots caused animals to swim faster (*z* = −3.05, *p* = .002, $R_{m}^{2}$ = .20), although the swimming speed varied considerably among individuals ($R_{c}^{2}$ = .93; Figure 4a). No significant change was observed at 10 knots (*z* = −.57, *p* = .568; Figure 4a). Porpoises tended to move further away from the boat track when approached by boats at both 10 and 20 knots, and there was only little variation among individuals (10 knots: t = −4.57, *p* < .001, $R_{m}^{2}$ = .12, $R_{c}^{2}$ = .19; 20 knots: t = −2.28, *p* = .023, $R_{m}^{2}$ = .05, $R_{c}^{2}$ = .05; Figure 4b). However, the probability that animals moved away from the boat track depended on the boat speed (*p* = .02, interaction term between log_10_(distance) and boat speed). Specifically, porpoises were more inclined to move away when approached at a speed of 10 knots (*z* = −2.37, *p* = .002, $R_{m}^{2}$ = .13, $R_{c}^{2}$ = .28; Figure 4c). Although most animals started moving away from the boat track when the boat was 100–200 m away, some animals did not move away till the boat was very close (Figure A3 in Appendix 1). Turning angles did not increase as the boat approached (*z* = −1.06, *p* = .289 for 10 knots; *z* = .11, *p* = .908 for 20 knots), and neither did the probability of diving deep (*z* = −2.20, *p* = .027 for 10 knots; *z* = −1.21, *p* = .226 for 20 knots). Additionally, porpoises did not breathe less (*z* = 1.88, *p* = .060 for 10 knots; *z* = 1.28, *p* = .200 for 20 knots).

### 3.2 Porpoise behaviour before and during exposure

**FIGURE 5:**
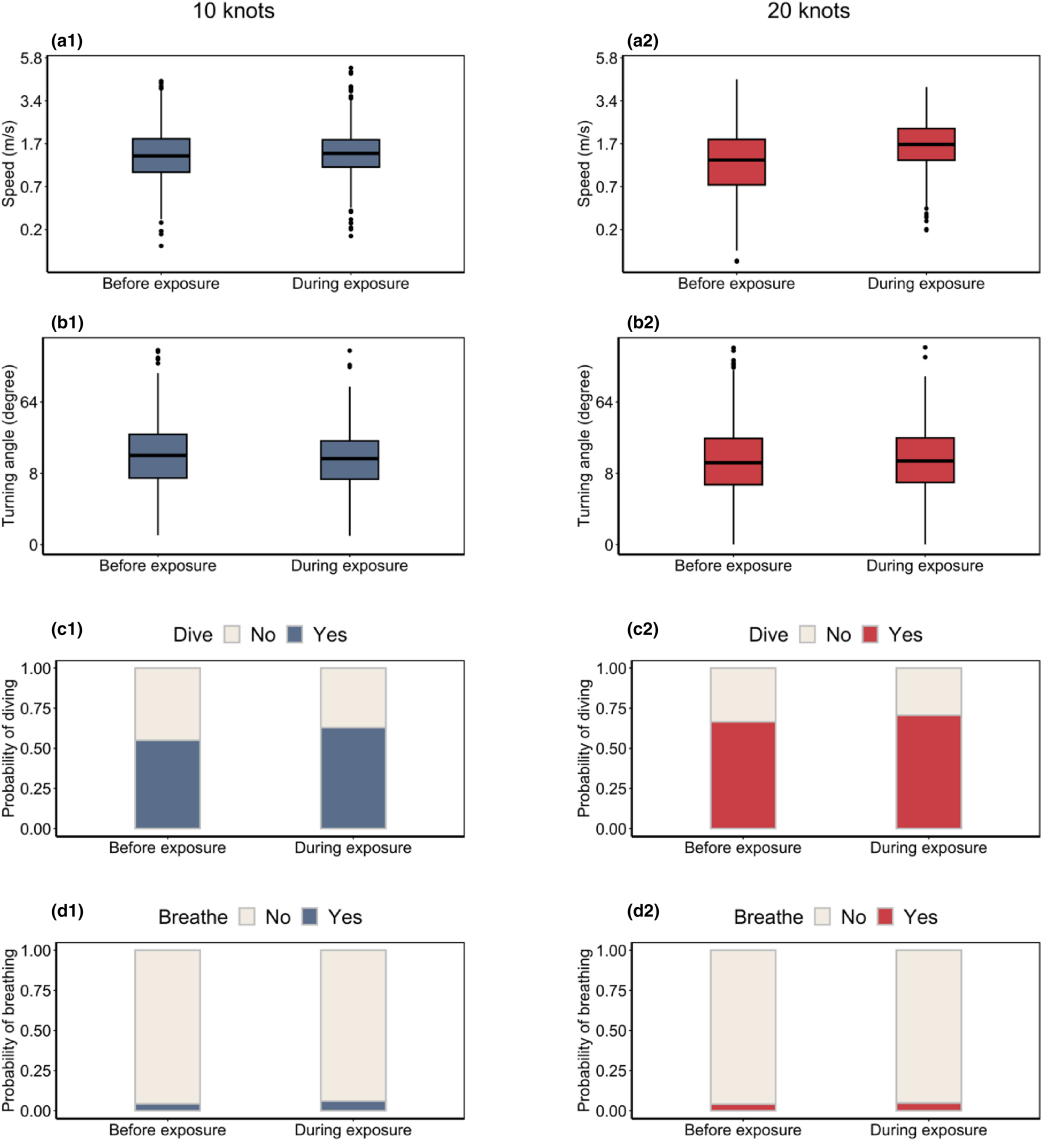
Porpoise behaviour before (phase 1) and during (phase 2) boat exposure, measured over a period of 1 min.

The porpoises' behaviour during the minute where the boat was closest did not, on the average, differ from their behaviour before the experiment started (their speed, absolute turning angles, and the probability of diving deep and of breathing, were the same; *p* > .025 for all variables and both boat speeds; Figure 5).

### 3.3 Time before resuming pre‐disturbance behaviour

**FIGURE 6:**
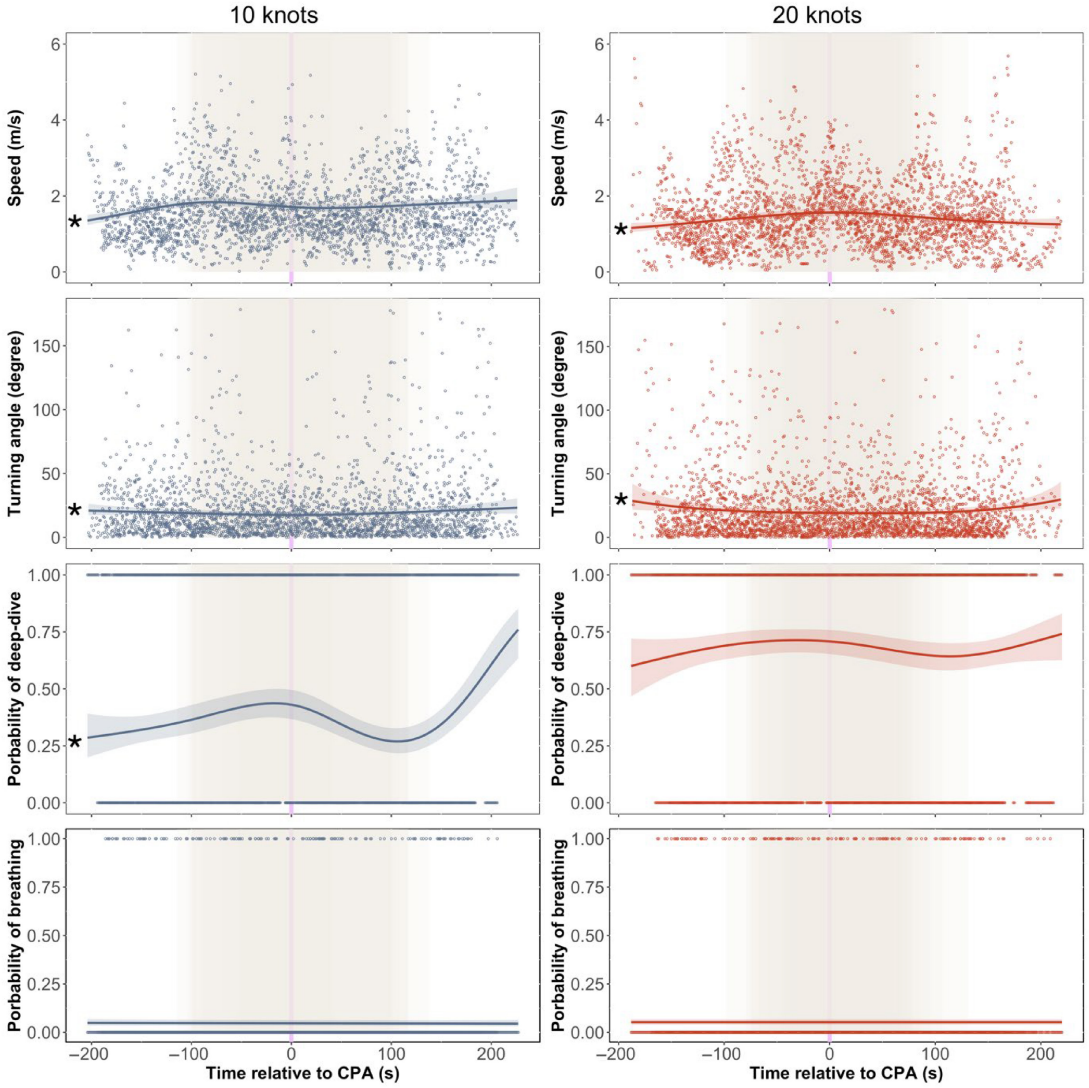
Observations (dots) and model estimates (smooth curves) of variations in porpoise behaviour during the exposure experiments (phases 1, 2 and 3). Shaded areas along smooth curves show 95% confidence intervals. The vertical purple lines represent the closest point of approach (CPA). The shaded light brown areas indicate the periods where the boat was in motion (i.e. phase 2). * denotes statistically significant models (*p* < .025).

Porpoise speeds varied over the course of the experiments (10 knots: *p* < .001, *R* ^2^ = .083; 20 knots: *p* < .001, *R* ^2^ = .17). This was particularly evident in the 20 knots experiments, where animals tended to move faster when the boat approached, and to slow down again <50 s after the boat had passed. When the boat approached at 10 knots, this trend was less clear (Figure 6). However, many animals moved as fast when the boat was not in motion as they did when the boat was nearby. Additionally, porpoises were more likely to dive deep as the boat approached at 10 knots, but to resume their normal behaviour shortly after it passed (*p* < .001, *R* ^2^ = .11). Nevertheless, no similar trend was observed at 20 knots (*p* = .09). Although the results presented above did not suggest that porpoises turned more steeply or that they were more likely to dive deep when approached by boats, the GAM analyses indicated that the porpoises' horizontal movements changed significantly in the course of the experiments (10 knots: *p* = .003, *R* ^2^ = .029; 20 knots: *p* < .001, *R* ^2^ = .028). However, time relative to CPA explained a very small proportion of the variation in this behaviour. The probability of breathing remained unchanged throughout the experiments for both speeds (*p* > .025 for both boat speeds).

### 3.4 Sound received from an approaching boat

**FIGURE 7:**
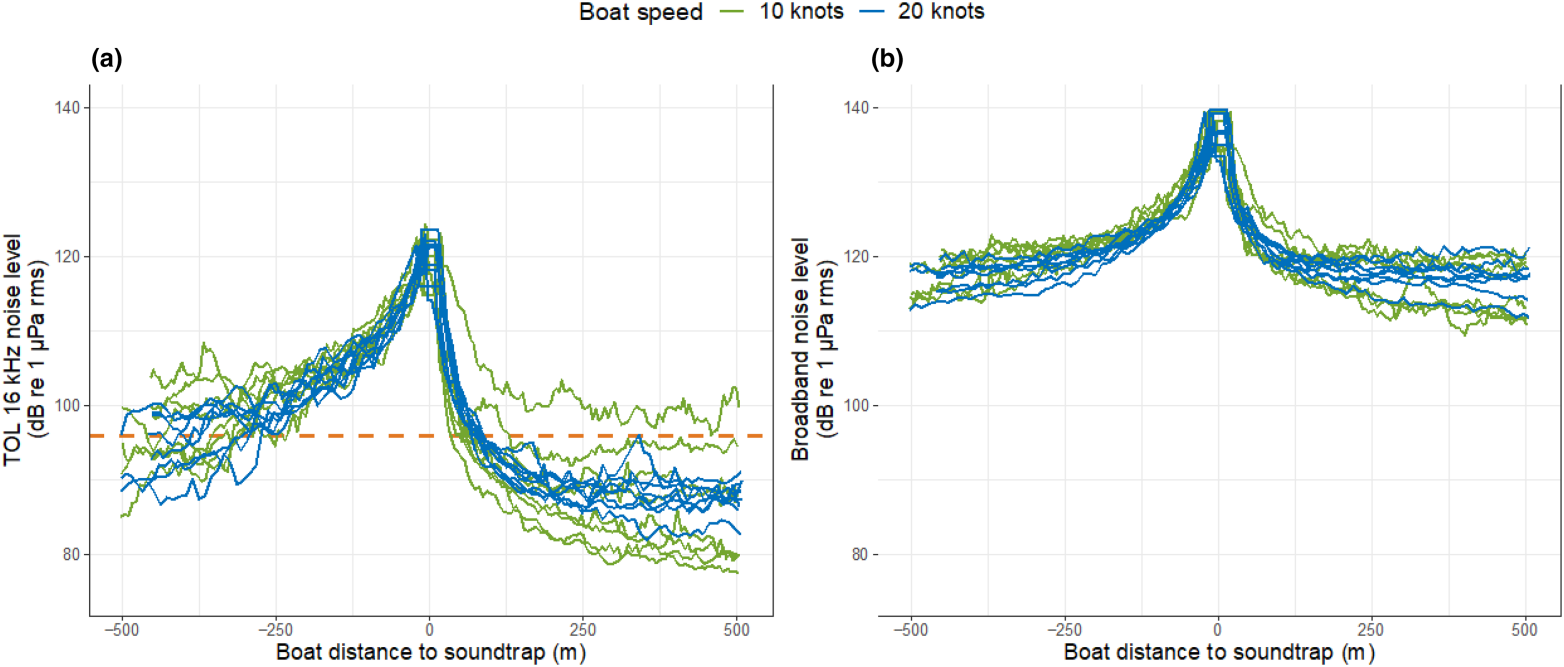
Received sound levels (1‐ws averages) measured within (a) 1/3 Octave (TOL) centred at 16 kHz and (b) broadband (0.1–150 kHz) from various distances of a boat approaching at 10 or 20 knots; negative distance values mean the boat was approaching the acoustic data logger. The dashed horizontal orange line indicates the threshold of porpoises' behavioural reactions to noise reported by Wisniewska et al. (2018). Shaded areas indicate distance ranges where most porpoise reactions appeared to take place, as based on raw data.

As the boat moved towards the CPA at 10 knots, the TOL 16 kHz sound level increased from approximately 85 to 125 dB while the broadband sound level (0.1–150 kHz) increased from 115 to 140 dB. The increase in sound level was similar at 10 knots (TOL 16 k: 28.1 ± 5.3 dB (mean ± SD); broadband: 22.8 ± 4.7 dB) and 20 knots (TOL 16 k: 29.3 ± 4.6 dB; Broadband: 23.0 ± 3.2 dB) for both sound frequency bands (Figure 7). The broadband sound level was about 15 dB higher than the TOL 16 kHz band level at CPA. At a speed of 10 knots, the mean absolute change in noise level per 10 s was 2.3 dB for broadband levels and 3.1 dB for 16 kHz TOL. Conversely, at 20 knots, the corresponding changes were 3.6 dB for broadband levels and 5.1 dB for the 16 kHz TOL. In the 10‐knot scenario, the most rapid change in sound levels occurred during the 10 s around CPA, where the mean changes in sounds reached 12.4 and 18.1 dB for broadband and 16 kHz TOL, respectively. In the 20‐knot recordings, these changes were even more pronounced; 11.9 dB per 10 s for broadband and 19.0 dB per 10 s for 16 kHz TOL.

Importantly, porpoises exhibited obvious reactions, including speeding up and moving away from the boat, when the approaching boat was within the range of 100–200 m. This coincided with a rapid rise in sound levels that started occurring from around 200 m with noise levels at 100–105 dB at 16 kHz TOL (Figures 4 and 7). After the boat had passed, the sound levels rapidly decreased (Figure 7).

## 4 DISCUSSION

Porpoises moved faster when approached by a recreational boat at 20 knots and were more likely to move away from the boat path when approached at 10 knots. In addition, they consistently increased their distance from the boat's path as it approached, irrespective of the boat's speed (Figure 4). These results do not entirely support our hypothesis; higher boat speeds elicited different behavioural reactions rather than stronger ones. Furthermore, strong behavioural changes were observed only when the boat approached in close proximity (<100–200 m; Figure 4). Once the boat passed, porpoises quickly resumed normal behaviour (<50 s; Figure 6), and their movements during the minute where the boat was closest did not differ significantly from their behaviour before the experiment started (Figures 5 and 6). These results indicate that the direct impact of the boat was brief, and that the behaviour observed for most of the animals during exposure was similar to their – often highly variable – behaviour before the experiment started.

Earlier research has suggested that either the absolute received noise level or rate of increase in received noise level may trigger porpoise responses to vessels (Wisniewska et al., 2018). In our study, noise measurements by the acoustic data logger independently of the drone experiments showed similar levels when the boat moved at 10 and 20 knots at a specific distance. This was the case both for TOL 16 kHz and broadband sound (Figure 7), suggesting that the difference in porpoise reactions to boats approaching at different speeds is due to the rate of change in noise level, rather than the noise level itself. Furthermore, animals generally speed up (in the 20 knots experiments) and moved away from the boat path when the boat was close (<100–200 m away; Figure 4a,b). At this distance sound had reached 100–105 dB at 16 kHz TOL, corresponding to a rapid increase in sound intensity (Figure 7). These results support earlier findings, that porpoises respond to noise levels exceeding 95–96 dB re 1 μPa at the TOL 16 kHz frequency band (Tougaard et al., 2015; Wisniewska et al., 2018). However, the observed noise level is below the threshold of 123 dB re 1 μ Pa at 0.25–63 kHz octave bands reported by Dyndo et al. (2015), possibly because the porpoises in their study were kept in a net pen, and had regularly been exposed to boat passages over 10 years. Thus, they could have become accustomed to boat noise.

We expected porpoises to turn more noticeably when approached by a boat, which would have resulted in less predictable movements. However, this was not what we observed. In a study of Black Sea harbour porpoises in Istanbul Strait, Turkey, porpoises tended to turn more when vessels were nearby (<200 m; Baş et al., 2017). A likely reason for this difference could be the fine resolution of our data, which was collected at 1‐second intervals. At this frequency, porpoises might not make significant turns every second that our analysis could detect. We also expected porpoises to be more likely to dive deep, as it has previously been reported for animals in the inner Danish waters (Frankish et al., 2023; Wisniewska et al., 2018), but this was not the case. One possible explanation is that the water was less than 7 m deep, which might limit the effectiveness of diving as an avoidance strategy. Additionally, we had expected animals to have a lower likelihood of breathing when the boat approached, thus allowing them to dive longer, but we did not observe any change in dive behaviour. However, we failed to follow seven porpoises during boat approaches as they dove too deep and did not resurface in the same area (10 knots: 4 instances; 20 knots: 3 instances). Overall our results indicate that boats can affect porpoise behaviour, but only when they are close to the boat, and for a short period of time.

The variability in how porpoises responded to the approaching boat highlights the complexity of their interactions with boats (Figure 4, Figure A3 and Table A1 in Appendix 1). This can be influenced by factors including proximity to the boat, the relative orientation of the porpoise to the boat track and the behavioural state of the porpoise at the time when the boat approaches. For example, harbour porpoises usually mate between July and August in Danish waters (Sørensen & Kinze, 1994), and two of the animals in our study were chasing each other and attempting to mate, which may have diverted their attention from the approaching boat. These observed differences in reactions illustrate the importance of studying a random sample of the population, rather than merely reporting an apparent change in behaviour for a few animals far from a vessel or other disturbances. Even though our data can presumably be considered a random sample of the population, it only consists of exposures in one high‐traffic boating area. Porpoises may respond more strongly to boats in areas where disturbances are less frequent.

Another reason why the studied porpoises did not respond to boats in the same way was that some of them appeared to protect their calves. Despite a general pattern of short‐term responses (Figure A3; Table A1 in Appendix 1), the six porpoises that were together with a calf either positioned themselves between their calves and the boat or stayed close to their calves while moving away from the boat. This suggests that porpoises perceive boats as threats, but perhaps a threat they only need to react to strongly if they need to protect a calf.

Our findings add valuable insights into how cetaceans respond to vessel disturbance. While porpoises in our study reacted similarly to those observed by Baş et al. (2017) and Oakley et al. (2017), other studies have documented porpoises to react to vessels over 1 km away (Palka & Hammond, 2001; Wisniewska et al., 2018). This variance underscores the necessity of employing diverse methods to assess the impact of vessels contextually, even within the same species. Moreover, some cetaceans respond negatively in ways that resemble those observed in this study. For example, bottlenose dolphins (*Tursiops truncatus*) have been observed to alter the way they move (Lemon et al., 2006; Marley et al., 2017; Nowacek et al., 2001) and southern resident killer whales (*Orcinus orca*) sometimes increase their swimming speed (Williams et al., 2009). The pattern of late reaction and quick recovery after boat passages we observed for porpoises has also been observed for Chilean dolphins (*Cephalorhynchus eutropia*; Ribeiro et al., 2005) and for bottlenose dolphins (Lemon et al., 2006). This short‐term reaction is likely a strategy to reduce energy expenditure. Some cetaceans appear entirely unaffected by close vessel encounters (Failla et al., 2004), and there are even examples of animals being attracted to vessels (Mesnick et al., 2002). This diversity in responses highlights that cetaceans differ in their sensitivity to vessel disturbances, and that they adopt different strategies to avoid them.

The widespread presence of recreational boats exposes cetaceans to high levels of disturbance. In Danish waters, where our study was conducted, recreational boats are often found in areas that are important harbour porpoise habitats (Hao & Nabe‐Nielsen, 2023). However, since the porpoises only appear to react to recreational vessels for a very short period when vessels are <200 m away, the population impact of this type of disturbances may be limited. Nevertheless, as porpoises are affected by a wide range of anthropogenic pressures, including sound from vessels, bycatch, chemical pollutants, and climate change (MacLeod et al., 2007; Nabe‐Nielsen et al., 2014; Pierce et al., 2008), the joint impact of the different pressures may influence population dynamics negatively (Gallagher et al., 2021; Nabe‐Nielsen et al., 2018). This emphasises the need for holistic assessments of the combined impacts of different stressors, and to do this, it is important to obtain field data of the impacts of each stressor in isolation. This requires an experimental setup, like the one we have used here, where we provide the first direct results on how harbour porpoises react to approaching vessels in Danish waters.

## AUTHOR CONTRIBUTIONS

**Xiuqing Hao:** Conceptualization (equal); data curation (equal); formal analysis (equal); investigation (equal); methodology (equal); software (equal); validation (equal); visualization (equal); writing – original draft (equal); writing – review and editing (equal). **Héloïse Hamel:** Conceptualization (equal); data curation (equal); investigation (equal); methodology (equal); writing – review and editing (equal). **Céline Hagerup Grandjean:** Investigation (equal); writing – review and editing (equal). **Ivan Fedutin:** Investigation (equal); writing – review and editing (equal). **Magnus Wahlberg:** Conceptualization (equal); data curation (equal); formal analysis (equal); methodology (equal); resources (equal); validation (equal); writing – review and editing (equal). **Caitlin Kim Frankish:** Conceptualization (equal); methodology (equal); validation (equal); writing – review and editing (equal). **Jacob Nabe‐Nielsen:** Conceptualization (equal); methodology (equal); supervision (equal); writing – original draft (equal); writing – review and editing (equal).

## CONFLICT OF INTEREST STATEMENT

The authors have no relevant financial or non‐financial interests to disclose.

## Data Availability

The raw data used for the analysis in this study, including porpoise locations, swimming states, boat locations and recorded boat noise levels, are accessible on Dryad (10.5061/dryad.q83bk3jq8). The R scripts used for conducting the analysis and calculating porpoise's avoidance behaviour are also available at the same location.

## APPENDIX 1

**TABLE A1:** Summary of porpoise metadata. Group size was determined as the number of porpoises at the beginning of each experiment.

Boat speed (knots)	ID	Group size	Identity
10	10_1	1	
	10_2	2	Mother
	10_3	1	
	10_4	2	Mother
	10_5	1	
	10_6	2	
	10_7	3	Mother
	10_8	1	
20	20_1	1	
	20_2	1	Mother
	20_3	3	
	20_4	2	Calf
	20_5	1	
	20_6	1	
	20_7	1	
	20_8	1	
	20_9	2	Mother

## References

[ece311433-bib-0001] Álvarez‐González, M. , Suarez‐Bregua, P. , Pierce, G. J. , & Saavedra, C. (2023). Unmanned aerial vehicles (UAVs) in marine mammal research: A review of current applications and challenges. Drones, 7(11), 667. 10.3390/drones7110667

[ece311433-bib-0002] Aubin, J. A. , Mikus, M.‐A. , Michaud, R. , Mennill, D. , & Vergara, V. (2023). Fly with care: Belugas show evasive responses to low altitude drone flights. Marine Mammal Science, 39(3), 718–739. 10.1111/mms.12997

[ece311433-bib-0003] Baş, A. A. , Christiansen, F. , Öztürk, A. A. , Öztürk, B. , & McIntosh, C. (2017). The effects of marine traffic on the behaviour of Black Sea harbour porpoises (*Phocoena phocoena relicta*) within the Istanbul Strait, Turkey. PLoS One, 12(3), e0172970. 10.1371/journal.pone.0172970 28296899 PMC5351841

[ece311433-bib-0004] Bejder, L. , Samuels, A. , Whitehead, H. , & Gales, N. (2006). Interpreting short‐term behavioural responses to disturbance within a longitudinal perspective. Animal Behaviour, 72(5), 1149–1158.

[ece311433-bib-0005] Brennecke, D. , Siebert, U. , Kindt‐Larsen, L. , Midtiby, H. S. , Egemose, H. D. , Ortiz, S. T. , Knickmeier, K. , & Wahlberg, M. (2022). The fine‐scale behavior of harbor porpoises towards pingers. Fisheries Research, 255, 106437. 10.1016/j.fishres.2022.106437

[ece311433-bib-0006] Brooks, M. E. , Kristensen, K. , Van Benthem, K. J. , Magnusson, A. , Berg, C. W. , Nielsen, A. , Skaug, H. J. , Machler, M. , & Bolker, B. M. (2017). glmmTMB balances speed and flexibility among packages for zero‐inflated generalized linear mixed modeling. The R Journal, 9(2), 378–400.

[ece311433-bib-0007] Brown, L. D. , Cai, T. T. , & DasGupta, A. (2001). Interval estimation for a binomial proportion. Statistical Science, 16(2), 101–133. 10.1214/ss/1009213286

[ece311433-bib-0008] Calenge, C. (2006). The package “adehabitat” for the R software: A tool for the analysis of space and habitat use by animals. Ecological Modelling, 197(3–4), 516–519.

[ece311433-bib-0009] Carreño, A. , & Lloret, J. (2021). Environmental impacts of increasing leisure boating activity in Mediterranean coastal waters. Ocean and Coastal Management, 209, 105693. 10.1016/j.ocecoaman.2021.105693

[ece311433-bib-0010] Chabot, D. , & Bird, D. M. (2015). Wildlife research and management methods in the 21st century: Where do unmanned aircraft fit in? Journal of Unmanned Vehicle Systems, 3(4), 137–155. 10.1139/juvs-2015-0021

[ece311433-bib-0011] Christiansen, F. , Rojano‐Doñate, L. , Madsen, P. T. , & Bejder, L. (2016). Noise levels of multi‐rotor unmanned aerial vehicles with implications for potential underwater impacts on marine mammals. Frontiers in Marine Science, 3, 277. 10.3389/fmars.2016.00277

[ece311433-bib-0012] Council Directive 92/43/EEC . (1992). Council Directive 92/43/EEC of 21 May 1992 on the Conservation of Natural Habitats and of Wild Fauna and Flora, 7.

[ece311433-bib-0013] Davenport, J. , & Davenport, J. L. (2006). The impact of tourism and personal leisure transport on coastal environments: A review. Estuarine, Coastal and Shelf Science, 67(1), 280–292. 10.1016/j.ecss.2005.11.026

[ece311433-bib-0014] Dyndo, M. , Wiśniewska, D. M. , Rojano‐Doñate, L. , & Madsen, P. T. (2015). Harbour porpoises react to low levels of high frequency vessel noise. Scientific Reports, 5(1), 11083. 10.1038/srep11083 26095689 PMC4476045

[ece311433-bib-0015] Egemose, H. D. (2021). Drone video measure . (1.1.1) [Computer software]. \https://github.com/egemose/DroneVideoMeasure

[ece311433-bib-0016] Failla, M. , Iñíguez, M. A. , Fernandez‐Juricic, E. , & Tossenberger, V. (2004). Effect of vessel traffic on Commerson's dolphin (Cephalorynchus commersonii) in Bahia San Julian, Patagonia, Argentina . International Whaling Commission, Scientific Committee (IWC‐SC) Report SC/56/WW7.

[ece311433-bib-0017] Fettermann, T. , Fiori, L. , Bader, M. , Doshi, A. , Breen, D. , Stockin, K. A. , & Bollard, B. (2019). Behaviour reactions of bottlenose dolphins (*Tursiops truncatus*) to multirotor unmanned aerial vehicles (UAVs). Scientific Reports, 9(1), 8558. 10.1038/s41598-019-44976-9 31189946 PMC6561960

[ece311433-bib-0018] Frankish, C. K. , von Benda‐Beckmann, A. M. , Teilmann, J. , Tougaard, J. , Dietz, R. , Sveegaard, S. , Binnerts, B. , de Jong, C. A. F. , & Nabe‐Nielsen, J. (2023). Ship noise causes tagged harbour porpoises to change direction or dive deeper. Marine Pollution Bulletin, 197, 115755. 10.1016/j.marpolbul.2023.115755 37976591

[ece311433-bib-0019] Gallagher, C. A. , Grimm, V. , Kyhn, L. A. , Kinze, C. C. , & Nabe‐Nielsen, J. (2021). Movement and seasonal energetics mediate vulnerability to disturbance in marine mammal populations. The American Naturalist, 197(3), 296–311. 10.1086/712798 33625969

[ece311433-bib-0021] Hao, X. , & Nabe‐Nielsen, J. (2023). Distribution and speed of recreational boats in Danish waters based on coastal observations and satellite images: Predicting where boats may affect harbour porpoises. Ocean and Coastal Management, 242, 106721. 10.1016/j.ocecoaman.2023.106721

[ece311433-bib-0022] Hermannsen, L. , Mikkelsen, L. , Tougaard, J. , Beedholm, K. , Johnson, M. , & Madsen, P. T. (2019). Recreational vessels without automatic identification system (AIS) dominate anthropogenic noise contributions to a shallow water soundscape. Scientific Reports, 9(1), 15477. 10.1038/s41598-019-51222-9 31664060 PMC6820791

[ece311433-bib-0023] Koh, L. P. , & Wich, S. A. (2012). Dawn of drone ecology: Low‐cost autonomous aerial vehicles for conservation. Tropical Conservation Science, 5(2), 121–132. 10.1177/194008291200500202

[ece311433-bib-0024] Lemon, M. , Lynch, T. P. , Cato, D. H. , & Harcourt, R. G. (2006). Response of travelling bottlenose dolphins (*Tursiops aduncus*) to experimental approaches by a powerboat in Jervis Bay, New South Wales, Australia. Biological Conservation, 127(4), 363–372.

[ece311433-bib-0025] Lennon, J. J. (1999). Resource selection functions: Taking space seriously? Trends in Ecology & Evolution, 14(10), 399–400. 10.1016/s0169-5347(99)01699-7 10481218

[ece311433-bib-0026] Lusseau, D. , Kindt‐Larsen, L. , & van Beest, F. M. (2023). Emergent interactions in the management of multiple threats to the conservation of harbour porpoises. Science of the Total Environment, 855, 158936. 10.1016/j.scitotenv.2022.158936 36152860

[ece311433-bib-0027] MacLeod, C. D. , Santos, M. B. , Reid, R. J. , Scott, B. E. , & Pierce, G. J. (2007). Linking sandeel consumption and the likelihood of starvation in harbour porpoises in the Scottish North Sea: Could climate change mean more starving porpoises? Biology Letters, 3(2), 185–188. 10.1098/rsbl.2006.0588 17251125 PMC2375924

[ece311433-bib-0028] Marley, S. A. , Salgado Kent, C. P. , Erbe, C. , & Parnum, I. M. (2017). Effects of vessel traffic and underwater noise on the movement, behaviour and vocalisations of bottlenose dolphins in an urbanised estuary. Scientific Reports, 7(1), 13437. 10.1038/s41598-017-13252-z 29044128 PMC5647363

[ece311433-bib-0029] Massicotte, P. , & South, A. (2023). Rnaturalearth: World map data from Natural Earth . R package version 0.3.3. https://CRAN.R‐project.org/package=rnaturalearth

[ece311433-bib-0030] Mesnick, S. L. , Archer, F. I. , Allen, A. C. , & Dizon, A. E. (2002). Evasive behavior of eastern tropical Pacific dolphins relative to effort by the tuna purse seine fishery . NOAA Admin Report LJ‐02‐30.

[ece311433-bib-0031] Morimura, N. , & Mori, Y. (2019). Social responses of travelling finless porpoises to boat traffic risk in Misumi West port, Ariake sound, Japan. PLoS One, 14(1), e0208754. 30601827 10.1371/journal.pone.0208754 PMC6314622

[ece311433-bib-0032] Nabe‐Nielsen, J. , Sibly, R. M. , Tougaard, J. , Teilmann, J. , & Sveegaard, S. (2014). Effects of noise and by‐catch on a Danish harbour porpoise population. Ecological Modelling, 272, 242–251. 10.1016/j.ecolmodel.2013.09.025

[ece311433-bib-0033] Nabe‐Nielsen, J. , Van Beest, F. M. , Grimm, V. , Sibly, R. M. , Teilmann, J. , & Thompson, P. M. (2018). Predicting the impacts of anthropogenic disturbances on marine populations. Conservation Letters, 11(5), 12563. 10.1111/conl.12563

[ece311433-bib-0034] Newey, W. K. , & West, K. D. (1986). A simple, positive semi‐definite, heteroskedasticity and autocorrelationconsistent covariance matrix. Econometrica, 55, 703–708.

[ece311433-bib-0035] Nowacek, D. P. , Christiansen, F. , Bejder, L. , Goldbogen, J. A. , & Friedlaender, A. S. (2016). Studying cetacean behaviour: New technological approaches and conservation applications. Animal Behaviour, 120, 235–244. 10.1016/j.anbehav.2016.07.019

[ece311433-bib-0036] Nowacek, S. M. , Wells, R. S. , & Solow, A. R. (2001). Short‐term effects of boat traffic on bottlenose dolphins, *Tursiops truncatus*, in Sarasota Bay, Florida. Marine Mammal Science, 17(4), 673–688.

[ece311433-bib-0037] Oakley, J. A. , Williams, A. T. , & Thomas, T. (2017). Reactions of harbour porpoise (*Phocoena phocoena*) to vessel traffic in the coastal waters of South West Wales, UK. Ocean and Coastal Management, 138, 158–169. 10.1016/j.ocecoaman.2017.01.003

[ece311433-bib-0038] Palka, D. L. , & Hammond, P. S. (2001). Accounting for responsive movement in line transect estimates of abundance. Canadian Journal of Fisheries and Aquatic Sciences, 58(4), 777–787. 10.1139/f01-024

[ece311433-bib-0039] Pierce, G. J. , Santos, M. B. , Murphy, S. , Learmonth, J. A. , Zuur, A. F. , Rogan, E. , Bustamante, P. , Caurant, F. , Lahaye, V. , Ridoux, V. , Zegers, B. N. , Mets, A. , Addink, M. , Smeenk, C. , Jauniaux, T. , Law, R. J. , Dabin, W. , López, A. , Alonso Farré, J. M. , … Boon, J. P. (2008). Bioaccumulation of persistent organic pollutants in female common dolphins (*Delphinus delphis*) and harbour porpoises (*Phocoena phocoena*) from western European seas: Geographical trends, causal factors and effects on reproduction and mortality. Environmental Pollution, 153(2), 401–415. 10.1016/j.envpol.2007.08.019 17905497

[ece311433-bib-0040] Pinheiro, J. , Bates, D. , DebRoy, S. , Sarkar, D. , & R Core Team . (2007). Nlme: Linear and nonlinear mixed effects models . R package version, 3(57), 1–89.

[ece311433-bib-0041] Ramos, E. A. , Maloney, B. , Magnasco, M. O. , & Reiss, D. (2018). Bottlenose dolphins and Antillean manatees respond to small multi‐rotor unmanned aerial systems. Frontiers in Marine Science, 5, 316. 10.3389/fmars.2018.00316

[ece311433-bib-0042] Rees, A. F. , Avens, L. , Ballorain, K. , Bevan, E. , Broderick, A. C. , Carthy, R. R. , Christianen, M. J. A. , Duclos, G. , Heithaus, M. R. , Johnston, D. W. , Mangel, J. C. , Paladino, F. , Pendoley, K. , Reina, R. D. , Robinson, N. J. , Ryan, R. , Sykora‐Bodie, S. T. , Tilley, D. , Varela, M. R. , … Godley, B. J. (2018). The potential of unmanned aerial systems for sea turtle research and conservation: A review and future directions. Endangered Species Research, 35, 81–100. 10.3354/esr00877

[ece311433-bib-0043] Ribeiro, S. , Viddi, F. A. , & Freitas, T. R. (2005). Behavioural responses of Chilean dolphins (*Cephalorhynchus eutropia*) to boats in Yaldad Bay, southern Chile. Aquatic Mammals, 31(2), 234–242.

[ece311433-bib-0044] Rizopoulos, D. (2022). GLMMadaptive: Generalized Linear Mixed Models using adaptive Gaussian quadrature . R package version 0.8‐5. https://CRAN.R‐project.org/package=GLMMadaptive

[ece311433-bib-0045] Sørensen, T. B. , & Kinze, C. C. (1994). Reproduction and reproductive seasonality in Danish harbour porpoises, *Phocoena phocoena* . Ophelia, 39(3), 159–176. 10.1080/00785326.1994.10429541

[ece311433-bib-0046] Sprogis, K. R. , Videsen, S. , & Madsen, P. T. (2020). Vessel noise levels drive behavioural responses of humpback whales with implications for whale‐watching. eLife, 9, e56760. 10.7554/eLife.56760 32539930 PMC7324156

[ece311433-bib-0047] Sveegaard, S. , Teilmann, J. , Tougaard, J. , Dietz, R. , Mouritsen, K. N. , Desportes, G. , & Siebert, U. (2011). High‐density areas for harbor porpoises (*Phocoena phocoena*) identified by satellite tracking. Marine Mammal Science, 27(1), 230–246. 10.1111/j.1748-7692.2010.00379.x

[ece311433-bib-0048] Tougaard, J. , Wright, A. J. , & Madsen, P. T. (2015). Cetacean noise criteria revisited in the light of proposed exposure limits for harbour porpoises. Marine Pollution Bulletin, 90(1–2), 196–208. 10.1016/J.MARPOLBUL.2014.10.051 25467877

[ece311433-bib-0049] Williams, R. , Bain, D. E. , Smith, J. C. , & Lusseau, D. (2009). Effects of vessels on behaviour patterns of individual southern resident killer whales *Orcinus orca* . Endangered Species Research, 6(3), 199–209. 10.3354/esr00150

[ece311433-bib-0050] Wilson, E. B. (1927). Probable inference, the Law of succession, and statistical inference. Journal of the American Statistical Association, 22(158), 209–212. 10.1080/01621459.1927.10502953

[ece311433-bib-0051] Wisniewska, D. M. , Johnson, M. , Teilmann, J. , Siebert, U. , Galatius, A. , Dietz, R. , & Madsen, P. T. (2018). High rates of vessel noise disrupt foraging in wild harbour porpoises (*Phocoena phocoena*). Proceedings of the Royal Society B: Biological Sciences, 285(1872), 20172314. 10.1098/rspb.2017.2314 PMC5829196 29445018

[ece311433-bib-0053] Wood, S. N. (2017). Generalized Additive Models: An introduction with R (2nd ed.). Chapman and Hall/CRC. 10.1201/9781315370279

